# Multi-Level Cell Properties of a Bilayer Cu_2_O/Al_2_O_3_ Resistive Switching Device

**DOI:** 10.3390/nano9020289

**Published:** 2019-02-19

**Authors:** Jonas Deuermeier, Asal Kiazadeh, Andreas Klein, Rodrigo Martins, Elvira Fortunato

**Affiliations:** 1 i3N/CENIMAT, Department of Materials Science, Faculty of Science and Technology, Universidade NOVA de Lisboa and CEMOP/UNINOVA, Campus de Caparica, 2829-516 Caparica, Portugal; j.deuermeier@campus.fct.unl.pt (J.D.); rfpm@fct.unl.pt (R.M.); emf@fct.unl.pt (E.F.); 2 Department of Materials and Earth Sciences, Technische Universität Darmstadt, Otto-Berndt-Straße 3, D-64287 Darmstadt, Germany; aklein@esm.tu-darmstadt.de

**Keywords:** resistive switching memories, multi-level cell, copper oxide, grain boundaries, aluminum oxide

## Abstract

Multi-level resistive switching characteristics of a Cu_2_O/Al_2_O_3_ bilayer device are presented. An oxidation state gradient in copper oxide induced by the fabrication process was found to play a dominant role in defining the multiple resistance states. The highly conductive grain boundaries of the copper oxide—an unusual property for an oxide semiconductor—are discussed for the first time regarding their role in the resistive switching mechanism.

## 1. Introduction

Two terminal resistive switches have motivated many studies on nanoscale data storage and neuromorphic applications due to their superior performance including high density, simple structure, fast programing, long retention, and low power consumption [1]. Conductive bridging random access memories (CBRAMs) are resistive switches which contain an electrochemically active metal electrode (Ag, Cu). It is widely accepted that programable metallization on the nanoscale within the switching medium causes bistable properties. To better control the localization and the diameter of conducting filaments (CFs) and improve the resistive switching uniformity and stability, bilayer devices (based on chalcogenides or oxides) consisting of a switching layer and a buffer layer were proposed some years ago [2,3]. Note that oxide bilayers are well compatible with complementary metal-oxide-semiconductor (CMOS) technology. 

Among numerous oxide-based switching materials, Cu_2_O and Al_2_O_3_ have advantageous properties such as being abundant, of low cost, having low processing temperatures, are environmentally friendly, and in the case of Cu_2_O, electrochemically active. Aluminum oxide is widely applied as a dielectric of thin film transistors [4,5], thus a resistive switching device based on Cu_2_O/Al_2_O_3_ may decrease the processing steps for circuits such as one transistor-one resistor cells (1T-1R). 

Various examples of resistive switching with a single Cu_2_O layer sandwiched between two electrodes have been reported. Starting in 1969, the disproportionation of Cu_2_O into Cu and CuO was considered to give rise to a copper filament [6,7]. In 1982 it was discussed that the Cu/Cu_2_O Schottky barrier was beneficial in creating reproducible switching events since it generates a region of comparatively high electric field [8]. However, the current blocking effect of a high barrier can also prevent switching from happening [9]. The most recent discussion of the switching mechanism concludes a filament of copper vacancies in the high conductance state and a Cu/Cu_2_O Schottky barrier in the low conductance state [10]. Others have reported multiple filaments of metallic copper; however, using devices with higher cell size and higher current than in the aforementioned work [11]. Note, that polycrystalline Cu_2_O was found to show highly conductive grain boundaries due to the presence of nanocrystalline CuO [12], causing the macroscopic conductivity to be corelated with the grain boundary density [13]. Another material in which defect segregation and increased conductivity in the grain boundary play a dominant role in resistive switching is HfO_2_ [14,15]. 

Thick insulators like Al_2_O_3_ and SiO_2_ do not conduct ions, but behave as electrolytes when sufficiently thin [16,17]. The result is a metallic filament growth from the inert electrode towards the active electrode. An opposite growth direction has also been reported both for Al_2_O_3_ [18] and SiO_2_ [19,20]. This mechanism appears to be favored when the metal is embedded as nanoclusters within the switching matrix. 

Two distinct types of low resistance states (LRS) were observed in a ZrO_2_/Cu-based memory, which were accessible by different set voltages. At low set voltages, an ionic filament based on defects in the switching matrix ZrO_2_ is activated, whereas at a higher set voltage a metallic filament based on copper is formed. The ionic filament is reset in a unipolar fashion, whereas the metallic one is bipolar [21]. In another bilayer memory device based on AlO*_x_*/WO*_x_*, multi-level cell (MLC) programing was accessed by controlling the current compliance (CC) during the set operation. The MLC approach decreased the processing cost of bits/cells. The conduction mechanisms in the LRS were found to be metallic for high CC, electron-hopping between metallic precipitates for intermediate CC and Schottky emission at the Al/AlO*_x_* interface for low CC and low temperature [22]. Another approach to control the metallic filament formation in a CBRAM device is to limit the supply of the reactive species by insertion of a buffer layer or by alloying the copper with another element [23,24,25]. 

This work presents the resistive switching behavior of Al_2_O_3_/Cu_2_O bilayer devices. Taking into account previously published knowledge on the structural and electrical inhomogeneity of polycrystalline Cu_2_O [12,13], as well as on the interface between Al_2_O_3_ and Cu_2_O [26], allows to clarify the roles of the individual layers in the resistive switching observed in the devices.

## 2. Materials and Methods 

**Figure 1:**
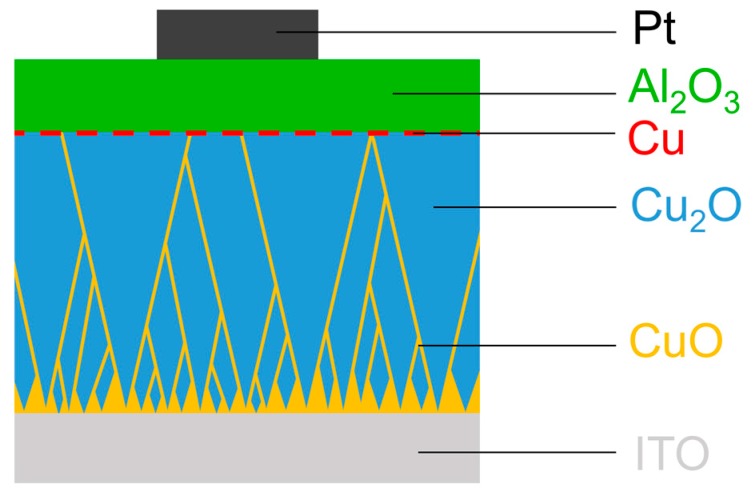
Device structure in cross-sectional view. The inhomogeneities in the copper oxide are schematically shown in orange (CuO-containing grain boundaries) and red (metallic copper at the interface between Al_2_O_3_ and Cu_2_O).

The device structure is schematically represented in Figure 1. Polycrystalline Cu_2_O (93 nm) was deposited by reactive magnetron sputtering from a metallic copper target (Kurt J. Lesker Company, Jefferson Hills, PA, USA) on commercial indium-tin oxide (ITO) on glass (Corning Inc., Corning, NY, USA). Subsequently, Al_2_O_3_ (16 nm) was deposited by atomic layer deposition (ALD) using trimethylaluminum (SAFC Hitech Ltd., Bromborough, UK) and water as precursors. Metallic copper was located between the copper oxide and the aluminum oxide layer as a consequence of the ALD process. Detailed information on the preparation method of these layers can be found in Reference [26]. As top contacts, sputter-deposited platinum (Kurt J. Lesker Company, Jefferson Hills, PA, USA) was used with a diameter of 100 μm, patterned by a shadow mask, using a commercial sputter-coater (Quorum Technologies Ltd., Lewes, UK). The current–voltage (I–V) characteristics of the ITO/Cu_2_O/Al_2_O_3_/Pt devices were measured at room temperature in air using a Keithley 4200-SCS semiconductor parameter analyzer (Keithley Instruments LLC, Cleveland, OH, USA) connected to a Janis ST-500 probe station (Janis Research Company LLC, Woburn, MA, USA). The bias was applied to the top electrode. The delay time until forming was measured under application of a constant voltage in sampling mode with a sampling interval of 0.5 s, using a measurement speed optimized for low noise and high accuracy (delay factor 1.3, filter factor 3, automatic A/D integration time setting). Temperature-dependent measurements were done in vacuum using liquid nitrogen for cooling, controlled by a Lake Shore 336 temperature controller (Lake Shore Cryotronics Inc., Westerville, OH, USA).

## 3. Results

**Figure 2:**
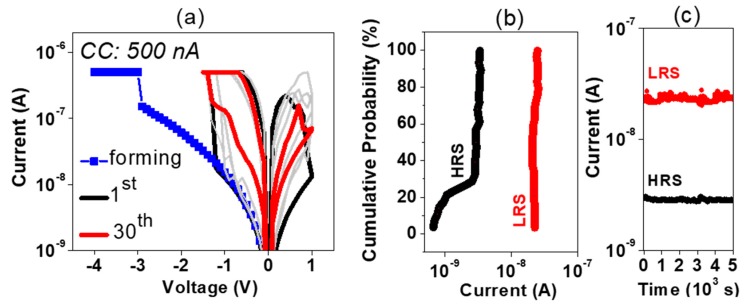
I–V sweeps of forming with a current compliance (CC) of 500 nA (blue squares) and 1st (black) and 30th (red) cycles, as well as a few intermediate cycles in grey (**a**), corresponding distribution of resistance states (**b**) and retention over 5 × 10^3^ s (**c**).

At first, the resistive switching template was tentatively formed by an electroforming process with low CC of 500 nA. Such a low current compliance allows for an extremely low power consumption of memory cells. The negative bias in voltage sweep mode was required to form the resistive switching memory device. The typical I–V behavior of the electroforming process is shown in Figure 2a. The memory cell reverted to the high resistance state (HRS) only by applying a positive voltage (reset) and the forming voltage was always higher than the required voltage for the set operation. The set and reset operating voltages are very low, <±1.5 V. An LRS/HRS ratio of 10^2^ was obtained at the first programming cycles, which was reduced and stabilized to ~10 after continued cycling. Initially, the HRS was as low as the pristine state (i.e., the electrical characteristic of the as-fabricated device), mainly due to the full rupture of initial narrow filament or a non-complete forming process. With further cycling, the I–V characteristics showed an enhancement of current rectification in the LRS, whereas the HRS current level increased after a few cycles, giving rise to an LRS/HRS margin below one order of magnitude. The self-forming during the initial cycles led to a selector-less device performance, which was highly stable, as represented by the distribution of the resistance states in Figure 2b and the retention data after the stabilization in Figure 2c.

**Figure 3:**
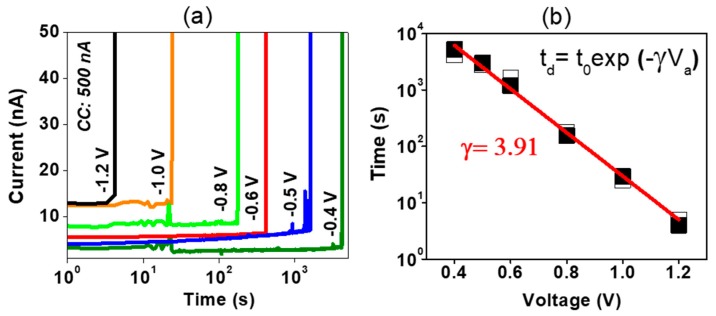
Current with respect to time under different constant voltage bias (**a**). Delay time to pre-forming with respect to voltage (**b**). Two individual measurements are shown by the filled and empty symbols. The exponential fit is shown in red.

In order to shed light on the pre-forming process (prior to the stabilization during cycling), the delay time *t*_d_ until pre-forming under constant voltage was measured for different voltages [27]. Each device was initially in the pristine state and a CC of 500 nA was used (see Figure 3a). Larger delay times were observed for smaller voltages. The delay time had an exponential dependence on voltage (see Figure 3b). A good fitting was obtained with *t*_0_ = 2.14 × 10^5^ s and γ = 3.91. The factor γ is referred to as a voltage acceleration parameter to a soft breakdown process [27] and higher values have already been reported for thinner films [28]. Similar results were reported for the breakage of a thin Al_2_O_3_ film [27]. This means, that the aluminum oxide plays a dominant role in the pre-forming process.

**Figure 4:**
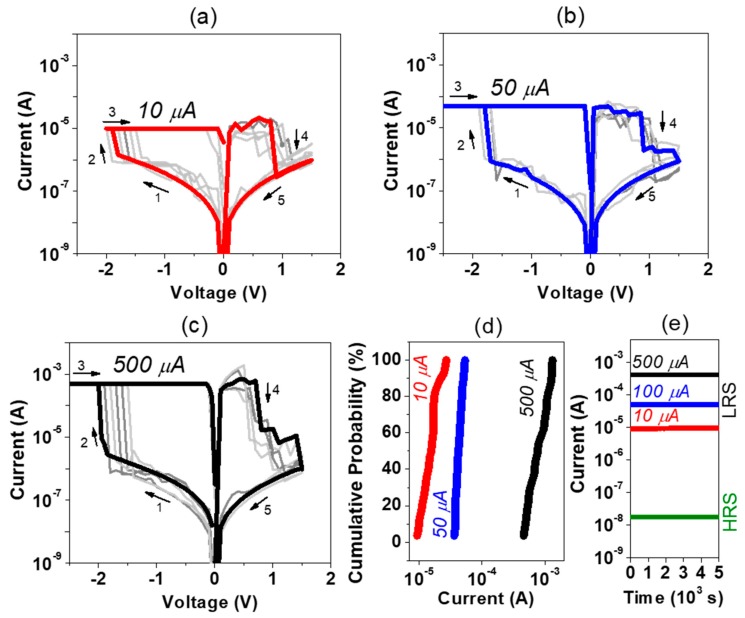
Typical I–V cycles of devices formed at CCs of 10 µA (**a**), 50 µA (**b**), and 500 µA (**c**), respectively, corresponding distribution of low resistance states (LRS) states (**d**) and retention over 5 × 10^3^ s (**e**).

A multi-level cell (MLC) operation can be obtained by changing the set and reset conditions. To modulate stable MLC states, a higher LRS/HRS ratio is required to obtain distinguishable conductance states for a long retention time. One way to increase the LRS/HRS ratio is to increase CC. Figure 4a–c show the typical I–V characteristics of different resistance states under application of CCs of 10 µA, 50 µA, and 500 µA, respectively. The distribution of different resistance states during sweeping cycles are shown in Figure 4d. The highest cycling stability was observed for a CC of 50 µA. All resistance states show no degradation in data retention over 5 × 10^3^ s, see Figure 4e. Note, that once the device sensed the 500 μA, the resistance states could not be well tuned for lower CC. The HRS was not dependent on the applied CC, suggesting an identical charge transport mechanism independent on the size of the filament.

**Figure 5:**
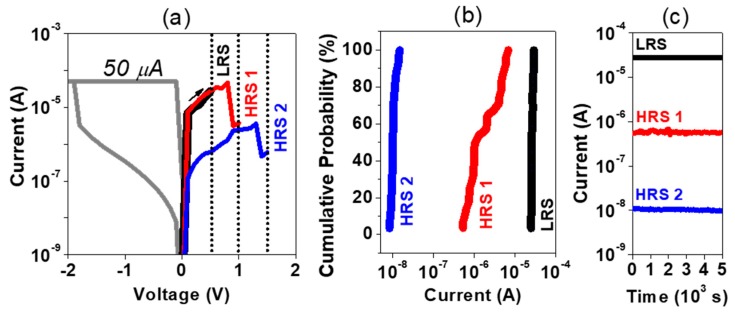
I–V sweeps of set at CC of 50 µA and reset to two distinct high resistance states (HRS), (**a**) with the corresponding distribution of resistance states (**b**) and retention over 5 × 10^3^ s (**c**).

Once the device is at a certain LRS, MLC can be also achieved by controlling the reset voltage. Figure 5a shows a typical I–V sweep under modulation of the reset voltage. Different conductance states are achieved after reset stop voltages of 0.5 V, 1 V, and 1.5 V. The conductance state distribution and the data retention of the MLC by controlling the reset voltages is shown in Figure 5b,c, respectively. Besides a good retention for all states, a higher spread of the conductance states was found for the HRS 1, compared to the LRS and HRS 2, which is discussed further below.

**Figure 6:**
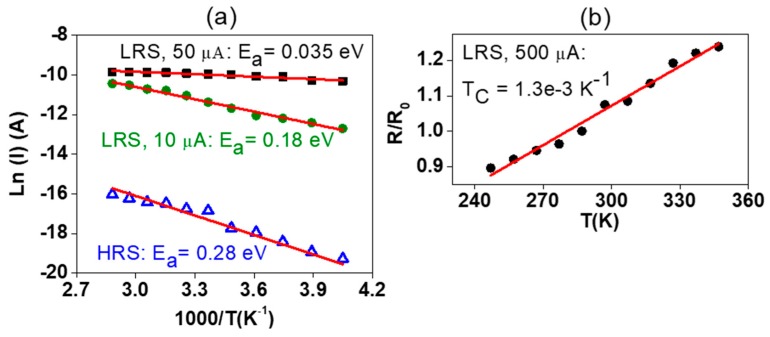
Arrhenius plots with corresponding fits (red lines) of the HRS (blue triangles) and the different LRS at CC of 10 µA (green circles) and 50 µA (black squares) (**a**), linear fit with respect to temperature (red line) of the LRS at CC of 500 µA (black circles) (**b**).

To investigate the MLC resistive switching behavior, the temperature dependence of the different states was studied, presented in Figure 6. Arrhenius plots of the HRS and the different LRS at CC 10 µA and 50 µA are shown in Figure 6a. A semiconducting behavior with good linear fits of the Arrhenius plots was obtained for the HRS and the LRS with a CC of 10 µA. The activation energies are *E*_a_ = 0.28 eV and *E*_a_ = 0.18 eV, respectively. When the CC was set for 50 µA, the current-temperature dependence still shows the semiconducting behavior, but no satisfactory linear fit was obtained, and the activation energy was too low to fulfill the conditions for the Boltzmann approximation. At high CC of 500 µA shown in Figure 6b, the conductance is decreased with the increase of temperature, which confirms a metallic conduction type. A positive temperature coefficient of 1.3 × 10^−3^ K^−1^ was obtained from fitting the results. 

## 4. Discussion

The mechanism of the conductive filament formation shall now be discussed based on the above presented results. The initial stages of forming are dominated by the soft breakdown of Al_2_O_3_. Since this was observed with a CC of only 500 nA, the conclusion of an initial forming in the Al_2_O_3_ holds also for higher CC. Note, that since a similar acceleration parameter has been reported for Al_2_O_3_ without the presence of a metallic species [27], we cannot be entirely certain about the nature of the filament in Al_2_O_3_. Due to the presence of metallic copper at the interface between Al_2_O_3_ and Cu_2_O (either as nanoclusters or as very thin film) [26], a filament formation from the interface with Cu_2_O towards the Pt electrode is likely [19,20], although the presented electrical data does not allow to be conclusive here. Independently of the filament growth direction, a counter reaction at the Pt electrode is required to fulfill the charge neutrality condition. Since electroforming was conducted in ambient air, moisture is the most probable reactant [29]. 

With the breakage of aluminum oxide, the resistance values of the MLC LRS were modulated by the filament formation in copper oxide. This conclusion is based on the activation energies given in Figure 6a, which closely resemble typical values of copper oxide (see detailed discussion further below). Metallic copper is not present close to the bottom electrode, so ions are oxidized, which are bound in the copper oxide lattice. Since water is typically reduced at the cathode [29], it is not considered for the anode reaction. Since the copper ions in CuO are fully oxidized (assuming stoichiometric CuO), the oxidation reaction must involve monovalent ions from the Cu_2_O grains. The corresponding anode reaction is written in Kroeger–Vink notation:

$${Cu}_{Cu}^{\times }\to Cu_{i}^{••}+V_{Cu}^{\prime }+e^{\prime }$$

Once the interstitial copper is formed, it becomes a mobile species and migrates towards the cathode, where it is deposited as metallic copper. Copper ions in copper oxide are highly mobile through a vacancy-assisted mechanism [30]. Besides the divalent copper ions, copper vacancies are created. These are the main acceptor defects in Cu_2_O [31]. Due to the formation of another phase (metallic copper), the cathode reaction is written as an electrochemical half-reaction.

$$Cu^{2+}+2e^{-}\to Cu$$

**Figure 7:**
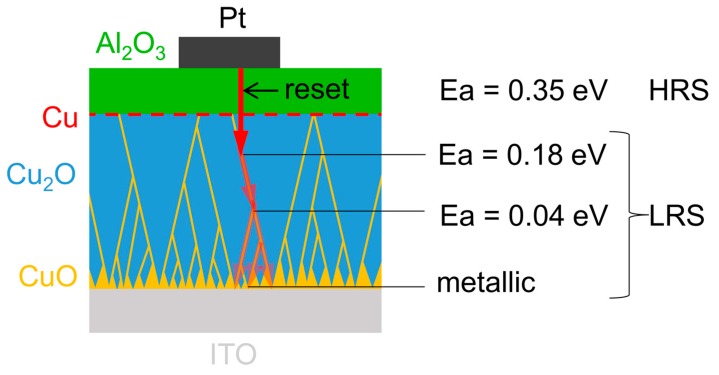
Schematic representation of the switching mechanism at different CCs. The varying extent of the filament inside the copper oxide with different CCs is illustrated by the red arrows.

The growth of the filament during forming in length and probably also in thickness depends on the CC [11]. This is schematically illustrated in Figure 7. Note that the grain boundaries of Cu_2_O are significantly more conductive than the grains due to the presence of CuO [12,13]. For this reason, most likely the filament formation involves the grain boundaries, which is considered in the following.

At lowest CC (500 nA), a Schottky barrier was observed in the positive polarity and the HRS was not stable during the initial cycles. The Schottky barrier clearly shows that copper is in electrical contact with Cu_2_O [32]. The instability shows that there are multiple competing highly conductive paths, which most likely correspond to the grain boundaries of Cu_2_O. Due to the instability during cycling, the forming of the memory is considered incomplete with CC = 500 nA. When the CC is increased to 10 µA, semiconducting filaments are observed. The activation energy is too low to be related to Cu_2_O [33]. A Schottky barrier is not observed anymore. This means that the copper filament is in direct contact with the copper oxide grain boundaries, since the grain boundary has electrical properties similar to CuO [13]. The absence of band bending in CuO causes the non-rectifying contact [34]. Compared to CC = 500 nA, an increased LRS stability is observed which is further increased with an even higher CC of 50 µA. With CC = 50 µA, the activation energy drops below 3kT (which the Boltzmann constant k and the temperature T), most likely due to an increased doping level of the CuO in the grain boundary of Cu_2_O and/or the progressed filament growth towards a region of higher grain boundary density close to the substrate interface (see Figure 7). Using a CC of 500 µA, the filament becomes metallic. The temperature coefficient of 1.3 × 10^−3^ K^−1^ is similar to the one observed in copper nanowires [35]. Hence, this LRS corresponds to a situation in which the metallic copper filament has reached the ITO bottom electrode during forming.

Concerning the reset process, it is important to note that Al_2_O_3_ has a lower thermal conductivity than Cu_2_O [36,37]. This means that the energy created due to Joule heating of the filament dissipated more efficiently in Cu_2_O than in Al_2_O_3_. This leads to a preferential rupture in the Al_2_O_3_, independent on the CC used during forming. The diffusion of copper is higher in Cu_2_O than in Al_2_O_3_ [38]. Hence, in case the filament in Al_2_O_3_ is based on metallic copper, it will be thinner in Al_2_O_3_ compared to Cu_2_O. The comparatively high activation energy in the HRS may be caused by the Al_2_O_3_ or potentially by a contribution of the Cu_2_O near-surface region, which is a region of comparatively high activation energy. Furthermore, as copper ions are released from the filament during reset, they can annihilate copper vacancies in the surrounding Cu_2_O grains, causing a decreased intrinsic doping level, thus an increase in activation energy.

With the reset occurring in or close to the Al_2_O_3_, the set process will also happen here. This means that the switching itself is of pure electrochemical metallization (ECM) type; however, with the difference of having mostly copper oxide as anode material and just a small amount of metallic copper (at the Cu_2_O/Al_2_O_3_ interface). This amount of metallic copper is modulated by the CC during forming, as described earlier. Hence, the copper supply at the Cu_2_O/Al_2_O_3_ interface can impact the switching characteristics similar to devices using Cu*_x_*Te_1-*x*_ as active electrode [24], which is reflected here by the presented results: low copper amounts (equivalent to low CC) compromise the filament stability (see Figure 2), whereas higher copper amounts (higher CC) require an increased reset voltage (see Figure 4a–c).

## 5. Conclusions

The resistive switching mechanism of a bilayer system of Cu_2_O and Al_2_O_3_ was investigated. The main objective of this work was to discriminate between the contributions of each individual layer and to discuss how they affect each other. The outcome features valuable indications for future bilayer device design. The observed multi-level operation was controlled by the current compliance. A transition from a semiconducting filament to a metallic filament with increasing current compliance was observed. For the filament growth in Cu_2_O, a dual mechanism is proposed, which involves a valence change in the copper oxide at the anode and a metallization reaction at the cathode. The likely preferential filament rupture during reset in the Al_2_O_3_ layer confines the switching event to Al_2_O_3_. Consequently, the supply of the active metal is conditioned by the CC-dependent filament growth in the copper oxide during forming, which directly impacts cycling stability.
